# ^68^Ga-DTPA Anti-HER2 positron emission tomography/CT successfully predicts the overexpression of human epidermal growth factor receptor in lung metastases from breast cancer

**DOI:** 10.1259/bjrcr.20160136

**Published:** 2017-05-06

**Authors:** Quetzali Gabriela Pitalúa-Cortés, Francisco O García-Pérez, Yolanda Villaseñor-Navarro, Fernando Ulises Lara-Medina, Juan Antonio Matus-Santos, Irma Soldevilla-Gallardo, Fany Iris Porras-Reyes, Victor Manuel Pérez-Sánchez, Héctor Aquiles Maldonado-Martínez, Wendy Pérez-Báez, Isabel Sollozo-Dupont

**Affiliations:** ^1^Deparment of Nuclear Medicine, Instituto Nacional de Cancerología, INCan, México; ^2^Department of Radiology and Imaging, Instituto Nacional de Cancerología, INCan, México; ^3^Deparment of Medical Oncology, Instituto Nacional de Cancerología, INCan, México; ^4^Deparment of Surgical Pathology, Instituto Nacional de Cancerología, INCan, México

## Abstract

Molecular identification of a metastatic tumour without the inconvenience of a biopsy and the time required for pathological characterization is possible using molecular imaging. Here, we present the case of a patient with breast cancer in whom ^68^Ga-diethylenetriamine pentaacetic acid anti-human epidermal growth factor receptor 2 positron emission tomography-CT was successfully employed to characterize the expression of human epidermal growth factor receptor 2 in metastatic sites.

## Background

Breast cancer is a major public health issue in low-income and middle-income countries.^1^ In these patients, it is not the primary tumour, but its metastases at distant sites, that is the main cause of death.^2^ On the other hand, among the most recurrent spots for metastatic expression as a consequence of breast cancer is the lung, predicting a poor prognosis with the cancer progression to this organ.^2–3^

Concerning pathogenesis, it is well known that human epidermal growth factor receptor 2 (HER2), when overexpressed, is associated with an aggressive form of breast cancer with a significantly shortened disease-free and overall survival.^4^ In fact, the determination of HER2 status is imperative in the primary breast cancer tissue to select an appropriate pharmacological intervention.^5^

Despite a biopsy being commonly required to provide the tissue diagnosis, as breast cancer progresses, collecting samples from metastases is not a routine practice.^6^ Therefore, therapeutic decisions for advanced disease are often based on the features of the primary tumour, neglecting evolutionary aspects of cancer progression that increase the risk of early death, such as immunophenotypic variations between primary and metastatic disease.^7–8^ For example, it is widely reported that breast cancer patients, who initially responded to endocrine therapy, are more vulnerable to develop *de novo* or acquired treatment resistance, notable here being the possible HER2 amplification.^8–9^

The infeasibility of a biopsy of metastatic sites underscores the need to investigate further non-invasive molecular techniques to support therapy decisions; here, we report a case of HER2-negative primary breast cancer in which ^68^Ga-diethylenetriamine pentaacetic acid (DTPA) anti-HER2 positron emission tomography (PET)/CT detects the overexpression of HER2 in lung metastases. This case is clinically relevant as the assessment of the HER2 status lacked a precise definition of positivity. Thus, radionuclide molecular imaging was especially important to guide pathology.

## Case report 

In August 2008, a 43-year-old female underwent a partial mastectomy with axillary lymph node dissection for right-sided breast cancer. Pathological diagnosis confirmed an infiltrating canalicular carcinoma (1.0 × 1.2 cm in diameter) as the primary tumour. Lymph node metastasis was observed in 6/34 harvested nodes. The disease was classified as stage IIIA according to the Union for International Cancer Control Tumour Node Metastasis classification for breast cancer(T1C N2 M0). A subsequent immunohistochemical examination revealed positive expression of hormone receptors in the primary tumour as well as the HER2 status as negative.

After surgery, the patient received four cycles of 5-fluorouracil, adriamycin and cyclophosphamide, followed by radiotherapy (60 Gy) and 12 cycles of paclitaxel. Once adjuvant chemotherapy was completed, the patient received goserelin and tamoxifen during 2 and 5 years, respectively.

**Figure 1.**
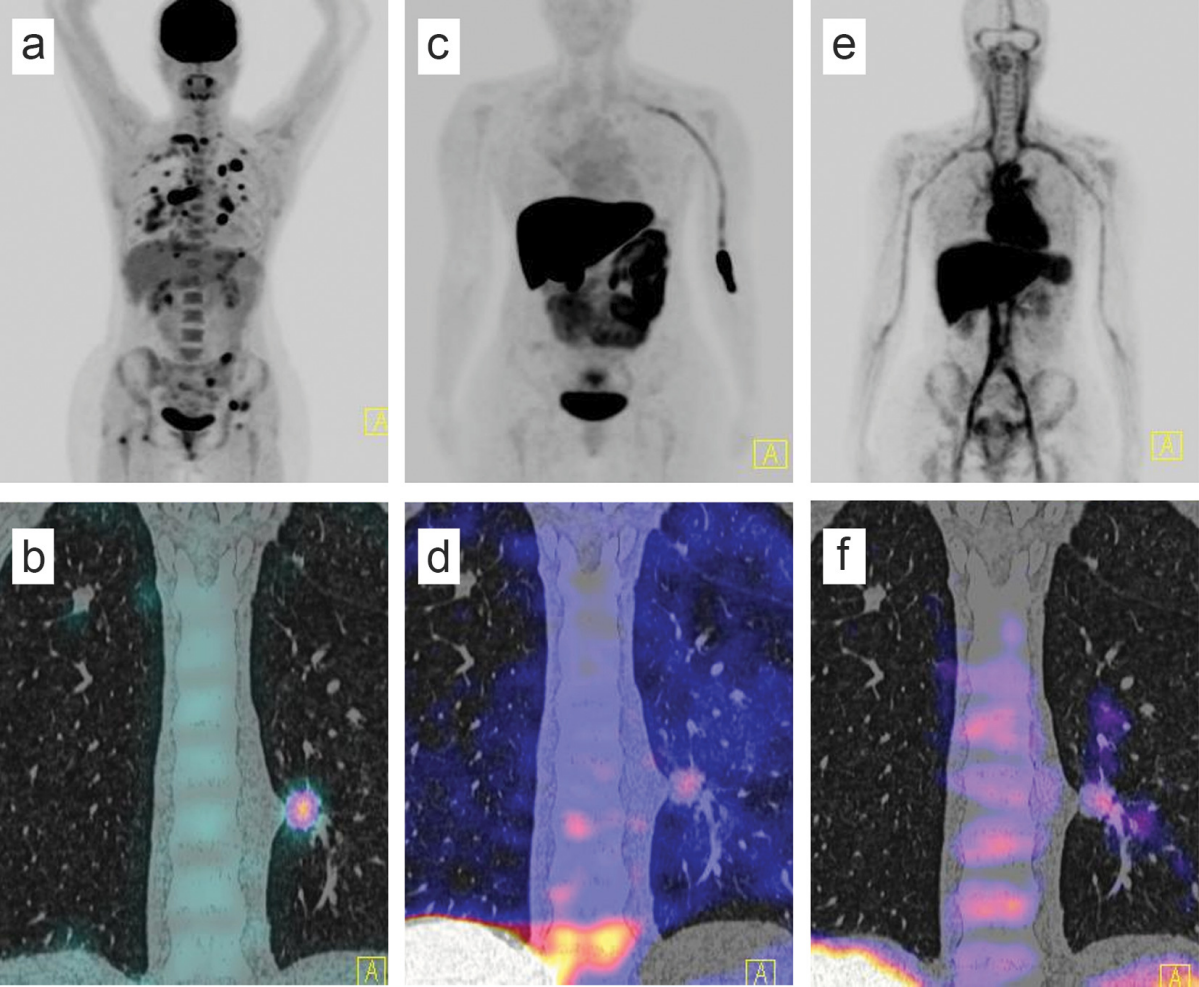
A 43-year-old woman with metastatic breast disease.^18^F-Fludeoxyglucose (FDG) PET/CT maximum intensity projection shows ^18^F-FDG-avid uptake in the lung, mediastinum and bone (a). Coronal PET/CT slice shows a 17 mm-pulmonary nodule localized at the posterior basal segment of the lower side of the left lobe with SUVmax of 10.7 (b). The patient was subject to another PET/CT study using ^18^F-fluoroestradiol (c). The maximum intensity projection shows focal uptake sites in the lung (d). Consistent with the metabolic response seen in panel (b), the same focus had an increased response by using ^68^Ga-DTPA anti-HER2 (SUVmax = 3.3) (e, f).

Six years after surgery, in response to a complaint of respiratory symptoms, the patient underwent a CT. The CT revealed a mediastinal nodal conglomerate, multiple lung nodules and right pleural effusion. Then, the patient was subjected to a PET/CT with 2-deoxy-2-[fluorine-18] fluoro-D-glucose (^18^F-FDG) PET/CT to evaluate the extent of the metastatic lesions. The PET/CT revealed increased ^18^F-FDG uptake in the lungs, bones and axillary lymph nodes (Figure 1a). The largest pulmonary nodule (17 mm), localized at the posterior basal segment of the left lower lobe, showed an SUVmax value of 10.7, whereas a mediastinal lymph node had an SUVmax of 3.9.

Owing to the aggressiveness of the disease, a phenotypic conversion at metastatic sites was suspected, including modification of the hormone receptor status (ER, PR). Thus, a parallel study using ^68^Ga-DTPA anti-HER2 and ^18^F-16α-17β-fluoroestradiol (^18^F-FES) was subsequently conducted (Figure 1b,c).

Metastatic tumours of the lungs and lymph nodes were visualized with both radiotracers, and the SUVmax values from the largest lesions were presented as follows: 3.3 for the lung lesion and 3.4 for lymph node using ^68^Ga-DTPA anti-HER2, and 3.2 and 3.3 for the lung and lymph node with ^18^F-FES. Thus, the resulting biopsy specimens were obtained from both metastatic lesions exhibiting the highest uptake to confirm the HER2 overexpression.

**Figure 2.**
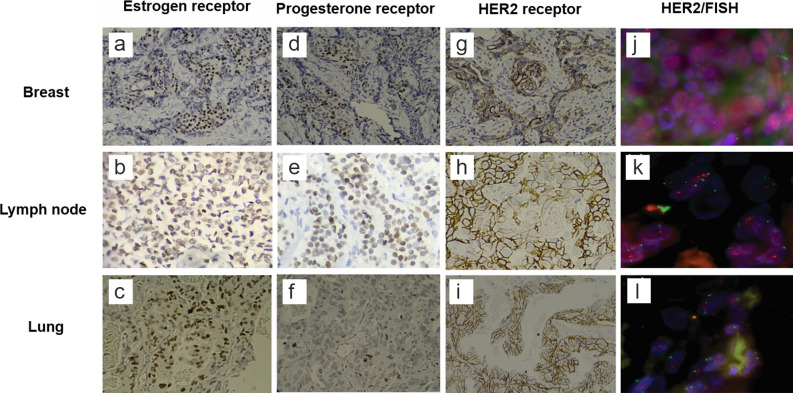
Images of the patient showing an infiltrating canalicular carcinoma with strong positive staining for oestrogen receptors in the breast (a), lymph node (b) and lung (c), with histological scores of 140, 120 and 130, respectively. Progesterone receptor expression with histological scores of 70, 40 and 5 in the breast, lymph node and lung (d–f) respectively. An indeterminate staining for HER2 analysis was visualized at the primary tumour (g) and both metastatic sites (h: lymph node; i: lung) by using immunohistochemistry (IHC; 2+ for all tissues). The expression of HER2 was corroborated by using fluorescence microscopy and in situ hybridization. The results were found as follows: Breast (j) not amplified (ERBB2:Cen17 ratio = 1.00) Nuclear in situ Hybridization (D17Z 1 × 2, ERBB2 × 2~17)[35]. Lymph node (k) amplified (ERBB2:Cen17 ratio = 4.08) Nuclear in situ Hybridization (D17Z 1 × 2, ERBB2 × 2~17)[60]. Lung (l) amplified (ERBB2:Cen17 ratio = 3.19) Nuclear in situ Hybridization (D17Z 1 × 2, ERBB2 × 2~17)[60].

Lastly, immunohistochemical examination revealed positive staining for hormone receptors in the breast (Figure 2a,d), lymph node (Figure 2b,e) and lung (Figure 2c,f), whereas HER2 was negative in the breast (Figure 2g) and indeterminate (2+) in both metastatic sites (Figure 2h,i). However, using fluorescence microscopy and in situ hybridization (FISH), HER2 overexpression was confirmed in the lymph node (4.18) as well as in the lung (3.19) (Figure 2k,l), whereas the primary tumour remained negative (Figure 2j).

## Materials and methods

### Preparation of ^68^Ga-DTPA Anti-HER2

Anti-HER2 (trastuzumab) was modified with cyclic diethylenetriamine pentaacetic acid (DTPA). The initial DTPA conjugation was performed at a 1:10 molar ratio with 100 mg of trastuzumab in 0.1 M sodium bicarbonate (pH 8). Ultrafiltration removed unbound DTPA. For radiolabelling of trastuzumab-DTPA, 50 mg of the conjugated monoclonal antibody and 2 ml of 0.1 sodium acetate (pH 5.5) were mixed with 185 MBq of ^68^Ga (2 ml, 0.1 M HCl) and incubated at room temperature for 10 min. The final product was sterilized by passing through a 0.22 μm syringe filter (Millex-GP syringe filter unit, Millipore).

### Administration of ^68^Ga-DTPA Anti HER2, ^18^F-FES and ^18^F-FDG

The patient underwent ^68^Ga-DTPA anti-HER2, ^18^F-fluoroestradiol (FES) and ^18^F-FDG PET/CT. The studies were performed on three separate days. The interval between these three scans was less than 7 days. ^68^Ga-DTPA anti-HER2 (185 MBq) was infused intravenously in 100 ml of saline for 10 min. ^18^F-FDG (237 MBq) was intravenously infused with Medrad® Intego PET Infusion System. Meanwhile, ^18^F-FES (222 MBq) was dissolved in 2 ml of saline solution and administrated in an intravenous bolus.

Only before the ^18^F-FDG PET/CT, the patient was requested to fast for at least 4 h. At the time of the tracer injection, a blood glucose level of 10 mmol l^–1^ was registered. The administration of ^68^Ga-DTPA anti-HER2 was closely monitored because of the risk of fever, chills and vomit.

The woman was kept lying comfortably in a quiet, dimly lit room during the time required for all procedures.

### PET-CT imaging

Images were obtained on a Biograph mCT 20 Excel PET/CT scanner (Siemens, Erlangen, Germany). Whole-body PET was performed 50–70 min after the injection of all tracers.

Low-dose CT was acquired using 120 kV, which was automated from 100–130 kV, a 512 × 512 matrix, a 50 cm field of view, 3.75 mm slice thickness and a rotation time of 0.8 s, extending from the vertex to the proximal thighs. Immediately after the CT acquisition, a whole-body PET was acquired. For each bed position (length 16.2 cm, overlapping scale 4.2 cm), we used 2 min acquisition time with a 50 cm field of view (matrix size 168 × 168). The emission data were corrected for randoms, scatter and decay. Reconstruction was conducted with an ordered subset expectation maximization algorithm with three iterations/12 subsets and Gauss-filtered to a transaxial resolution of 6 mm at full-width at half-maximum. Attenuation correction was performed using the low-dose non-enhanced CT.

### Image and pathological analysis

A workstation Multimodality Workplace (Siemens, Erlangen, Germany) providing multiplanar reformatted images was used for image display and analysis. The SUVmax of whole-body tumours were measured with the isocontour tool of the TrueD Syngo software (Siemens, Erlangen, Germany) with manual threshold adjustment. SUVmax was calculated with the radioactivity measured from an image acquired at the time, decay corrected to *t *= 0 and converted from a volume to a mass-based unit via the factor 1/(1 g ml^–1^), the injected dose at *t *= 0 and the body weight on the day of imaging.

Also, the correlation of ^18^F-FES uptake (SUVmax) with ER expression was measured by H-score. The *H-score or histo-score* is a method of assessing the extent of nuclear immunoreactivity, applicable to steroid receptors.^10^ The score was obtained by the formula: 3 × percentage of strongly staining nuclei + 2 × percentage of moderately staining nuclei + percentage of weakly staining nuclei, given a range of 0 to 300. When the H-score was ≥1%, positive cells to steroid receptors was confirmed.

^68^Ga-DTPA anti-HER2 uptake was compared with HER2 expression measured by immunohistochemistry (IHC) (positive or negative), ordering FISH only when the IHC results did not clearly show whether the cells were HER2-positive or negative. FISH results were presented as a quantitative score of the level of gene amplification, and the results were expressed as a ratio of the number of HER2 gene copies per number of chromosome 17 copies.^11^ The ratio was considered abnormal when it was greater than 1.8 (FISH+).

Tissue samples were collected 3 days after all imaging studies.

## Discussion

The data obtained from this clinical case show that ^68^Ga-DTPA anti-HER2 has the capability to identify HER2-expressing metastatic lesions from breast cancer. Importantly, HER2 overexpression was foreseen in lymph nodes and lung metastases by molecular imaging when the IHC analysis was indeterminate for both metastatic sites. In spite of ^18^F-FDG showing the highest uptake, ^68^Ga-DTPA anti-HER2 provided unique information of metastasis-specific biomarker status.

The origin of the moderate uptake of ^68^Ga-DTPA anti-HER2 remains to be determined, but may be related to low levels of HER2 expression.^12^ In any case, we assume that the volume of air contained in lungs enhanced the intrinsic tissue contrast, favouring the rapid visualization of metastatic lesions (40 min after ^68^Ga-DTPA anti-HER2 injection). However, it is important to note that the liver and heart had an intense radiotracer background activity (^68^Ga-DTPA anti-HER2 SUVmax = 9.45 and 8.7, respectively) that might obscure metastases, increasing the chance of getting false-negative in these organs.

Given the absence of liver and heart metastases, background activity was not a problem in the present case. Nevertheless, the possibility of missed lesions in a tissue that normally clears background activity over time (60–90 min post injection, such as the liver) has favoured the use of radioisotopes with a half-life longer than the half-life of ^68^Ga (t1/2 = 68 min). For instance, other research groups have developed PET/CT imaging strategies using the full therapeutic antibody trastuzumab labelled with ^89^Zr or ^64^Cu to identify HER2 overexpression.^13–16^ This approach, however, has an important disadvantage related to slow blood clearance, resulting in a late imaging time point of 1–2 d (^64^Cu) or 4–5 d (^89^Zr) after tracer injection, a long scanning time of up to 1 h and a high radiation burden of 12 mSv (^64^Cu) or 18 mSv (^89^Zr) for the patient.^12^ Additionally, ^89^Zr and ^64^Cu are produced by expensive accelerators or reactors, contrary to the possible greater production and availability of ^68^Ga.^17^

On the other hand, the slow blood clearance has been solved using fully functional segments of trastuzumab, dubbed “Nanobodies or Affibodies,”^18^ coupled with different radiotracers including ^68^Ga. Several studies demonstrated that those trastuzumab fragments found and bound HER2 receptors within the first hours after administration^12,19–21^ and this, together with rapid blood clearance, allowed imaging of tumours as early as 30–60 min after injection.^22^ For example, by comparing the high-contrasted images of HER2-binding Affibodies –025 and -002 with images obtained using the full antibody trastuzumab, the former are obtained in a shorter time with a significant reduction of the background activity within the liver.^12,19–24 ^

Furthermore, the present case indicates the possibility that an initially HER2-negative breast cancer can become HER2 positive following treatment. Multiple mechanisms are proposed to explain the HER2 conversion, including de novo heterogeneity and the formation of different subclones that can metastasize.^25–26^ Importantly, several authors reported that if during disease progression, a patient acquires HER2 positivity, the survival rate may decrease because of the possibility of resistance to treatment.^26–28^ Therefore, the reassessment of HER2 status using non-invasive PET/CT instead of performing a biopsy is now a key component of diagnosis, with the hope, when possible, of optimizing treatment in patients who could profit from trastuzumab or other therapeutic approaches targeted against HER2.^29–31^

Finally, according to pathologist guidelines for HER2 testing, IHC and FISH are equally reliable, having the same *precision *if properly carried out.^32^ Nevertheless, we consider that the difficulty in interpreting the 2+ IHC not only supports the utility of FISH but also reinforces clinical imaging using ^68^Ga-DTPA anti-HER2 PET/CT to guide pathology.

In summary, this report corroborates that ^68^Ga-DTPA anti-HER2 PET/CT is useful in characterizing changes in tumour expression of HER2 across breast cancer progression. However, more research is needed to expand the clinical utility of this radiotracer.

## Conclusions

This research supports the feasibility of using ^68^Ga-DTPA anti-HER2 for evaluating HER2 overexpression in metastatic breast cancer, demonstrating that the development of a pathology using *in vivo* molecular imaging may overcome limitations linked to conventional diagnostic studies of primary tumours.

## Learning points

1. The acquisition of tissue from metastatic deposits is not routine practice. Thus, following the development of distant metastases in breast cancer patients, systemic treatment selection depends on the tissue characteristics of the primary tumour.
2. Given that the expression of hormone receptors and HER2 of the primary breast cancer and its corresponding distant metastatic sites are often dissimilar, this can lead to an inappropriate choice of systemic treatment.
3. A potential mapping of hormone receptors and HER2 expression using non-invasive PET imaging analysis is presented here, demonstrating that radiotracers labelling specific receptors are important for exploring immunophenotypic variations in breast cancer metastatic disease.
4. In our opinion, in the future, advances in systemic therapy for breast cancer patients might depend, at least in part, on using specific PET-radioligands.

## Consent

Written informed consent for the case to be published (including images, case history and data) was obtained for publication of this case report.
